# Environmental Alterations during Embryonic Development: Studying the Impact of Stressors on Pluripotent Stem Cell-Derived Cardiomyocytes

**DOI:** 10.3390/genes12101564

**Published:** 2021-09-30

**Authors:** Federica Lamberto, Irene Peral-Sanchez, Suchitra Muenthaisong, Melinda Zana, Sandrine Willaime-Morawek, András Dinnyés

**Affiliations:** 1 BioTalentum Ltd., H-2100 Gödöllő, Hungary; federica.lamberto@biotalentum.hu (F.L.); suchitra.polgari@biotalentum.hu (S.M.); melinda.zana@biotalentum.hu (M.Z.); 2 Department of Physiology and Animal Health, Institute of Physiology and Animal Health, Hungarian University of Agriculture and Life Sciences, H-2100 Gödöllő, Hungary; 3 Faculty of Medicine, University of Southampton, Southampton SO16 6 YD, UK; I.Peral-Sanchez@soton.ac.uk (I.P.-S.); S.Willaime-Morawek@soton.ac.uk (S.W.-M.); 4 HCEMM-USZ Stem Cell Research Group, Hungarian Centre of Excellence for Molecular Medicine, H-6723 Szeged, Hungary; 5 Department of Cell Biology and Molecular Medicine, University of Szeged, H-6720 Szeged, Hungary

**Keywords:** Developmental Origins of Health and Disease (DOHaD), cardiovascular diseases (CVDs), pluripotent stem cells (PSCs), cardiomyocytes differentiation, environmental factors, epigenetics

## Abstract

Non-communicable diseases (NCDs) sauch as diabetes, obesity and cardiovascular diseases are rising rapidly in all countries world-wide. Environmental maternal factors (e.g., diet, oxidative stress, drugs and many others), maternal illnesses and other stressors can predispose the newborn to develop diseases during different stages of life. The connection between environmental factors and NCDs was formulated by David Barker and colleagues as the Developmental Origins of Health and Disease (DOHaD) hypothesis. In this review, we describe the DOHaD concept and the effects of several environmental stressors on the health of the progeny, providing both animal and human evidence. We focus on cardiovascular diseases which represent the leading cause of death worldwide. The purpose of this review is to discuss how in vitro studies with pluripotent stem cells (PSCs), such as embryonic and induced pluripotent stem cells (ESC, iPSC), can underpin the research on non-genetic heart conditions. The PSCs could provide a tool to recapitulate aspects of embryonic development “in a dish”, studying the effects of environmental exposure during cardiomyocyte (CM) differentiation and maturation, establishing a link to molecular mechanism and epigenetics.

## 1. Introduction

The significant increase of chronic diseases (e.g., diabetes, obesity, some cancers, cardiovascular diseases, neuronal disorders) is attributed more frequently to the influence of environmental factors, which have a pivotal role in disease aetiology. Previously, it was considered that the foetus in the uterus was free from damages caused by external agents (environment). However, it is now clear that exposure to different environments before birth plays a significant role in the origin of these diseases. Scientists are now increasingly focusing on the understanding of the hypothesis known as the Developmental Origins of Health and Disease (DOHaD) [1,2]. The DOHaD hypothesis has significant implications for understanding the epidemiology of non-communicable diseases (NCDs), especially those including the onset of cardiovascular, metabolic and neurological disorders [3,4]. One of the main goals of DOHaD-related research is to develop and evaluate interventions to improve health and prevent diseases that can occur at different stages of an individual life, e.g., gestation, childhood or adulthood [5]. 

To date, it is known that altered conditions during the periconceptional (PC) period of gamete maturation and early embryonic development have long-lasting effects on the health of the progeny. In other words, if the embryo is exposed to a hostile uterine environment it responds with adaptation to survive, however, in the long-term, this can lead to irreversible changes in development, structure and function of some tissues and vital organs [1,2]. Nonetheless, the exact mechanism of causes (i.e., cellular, metabolic and/or physiological alterations) behind this hypothesis are still poorly understood [2]. Epigenetic changes have a pivotal role and add to the complexity in the study of developmental programming [6,7,8]. 

The PC period might be a programming period of high susceptibility to maternal diet which can affect lifelong health in the offspring. This PC sensitivity may reflect how the number of totipotent and pluripotent cells in the embryo are modified by the extensive epigenetic restructuring that characterises this period. These changes in environmental conditions cause the embryo to optimise its future development program, resulting in a form of plasticity related to the concept of predictive adaptive responses in DOHaD [9].

Moreover, studies in animal models have shown that the PC environment influences subsequent development programming in mammalians [10,11,12]. Not only external environmental factors can influence the development of the embryo, but internal factors and the environment in which the embryo is located play also a role. Small changes in the environment (for example, changes in diet or exposure to toxins) or changes in the mother’s homeostasis, can affect the uterine environment, changing the uterine fluid composition (i.e., amino acids) affecting the embryo and its epigenetic status, and this can also result in changes later in life [10]. The uterine fluid plays an important role in the pregnancy [13], secreting or transporting bioactive substances that regulate the uterine preparation for the embryo implantation and development [14]. 

Previous studies have suggested that environment changes during the pre-implantation period affect the offspring, but it is also the case with changes before and after the pre-implantation period; for example, mothers who are exposed to a diet with an excess of fatty acids before conception and during pregnancy present structural changes in the thickness and surface of the placenta [15]. These changes may lead to a reduction in blood flow in the placenta, which may contribute to the development of diseases in the offspring (for example, in the immune system or cardiovascular system) [16]. 

Much of the evidence underpinning the DOHaD concept has been obtained from animal models and observational human studies [1,17,18,19]. Nevertheless, cellular models could also underpin the research in the DOHaD area. Indeed, the in vitro models allow to assess the functional properties of tissue and to study the stages of tissue development [20]. To that end, it is possible to manipulate pathways and mechanisms in order to consider additional downstream implications and to assess therapeutic interventions [21]. 

In this review, we provide an overview of the current studies recapitulating the DOHaD hypothesis, giving a general description of the human clinical evidence and the in vivo models used in this research. Our aim is to highlight the use of pluripotent stem cells (PSCs), including embryonic and induced pluripotent stem cells (ESC, iPSC), as a tool for modelling non-genetic cardiovascular diseases and for studying the effects of several stressors on cardiomyocytes (CMs) differentiation and maturation. 

## 2. The DOHaD Concept and Non-Communicable Diseases (NCDs)

The DOHaD concept explains that the late-onset diseases may stem from events originating in utero, as initially studied in the 1980s by Barker and colleagues [22,23]. Subsequent studies in this field ultimately led to DOHaD or “Barker’s hypothesis” [24]. Scientific evidence showed that offspring worldwide exhibit future disease risks associated with different exposures of their parents before and during the pregnancies, including chemical, nutritional and environmental stressors or with other conditions like reproductive failure, adverse pregnancy outcome, diabetes, obesity and assisted reproductive technologies (ART) [1]. Emerging data from animal and human studies revealed that the time around conception is crucial in the DOHaD concept. The PC period represents a window of a few weeks in humans when cells of the embryo are directly exposed to stressors that make them vulnerable to alterations in epigenetic, cellular, metabolic and/or physiological mechanisms [2].

During the last thirty years, scientists have focused on the mechanisms at the basis of NCDs in the DOHaD field due to the high risk of mortality that these diseases account for worldwide. According to World Health Organization (WHO), NCDs such as cardiovascular diseases, diabetes and some forms of cancer accounted for about 63% of all deaths globally in 2008 [25]. Nowadays, this number is even higher and accounts for 41 million people (more than 70% of all death world-wide) who die every year prematurely, between ages 30 and 70 years, due to heart attacks, stroke, cancer, chronic respiratory diseases, diabetes or mental disorders [26]. The most critical issue is that, initially, NCDs were regarded as problems primarily associated with the elderly, but to date, it is well known that these conditions also affect large numbers of younger people in low and middle-income countries [3,27].

**Figure 1:**
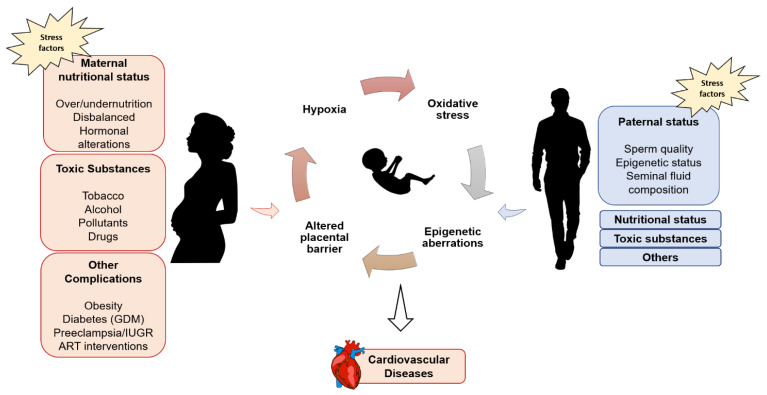
Maternal and paternal exposure to stress factors can perturb the foetal status and predispose to the onset of NCDs, such as cardiovascular diseases. Pregnancy environment contributes significantly to the newborn development, and numerous stressors have long-lasting effects on the health of the progeny. IUGR: intra uterine growth restriction, GDM: gestational diabetes mellitus.

Multiple developmental factors affect the health of the progeny, such as an unbalanced parental diet, smoking and alcohol consumption, exposure to toxins or pollutants and ART interventions (Figure 1). Nutrition and metabolic diseases (such as diabetes and obesity) are perhaps the most frequently investigated conditions for their susceptibility linked to the foetal period exposure [2,3,28]. Early interventions on the mother and/or infant represent the best preventive measures, and even during adolescence interventions are likely to be effective at counteracting NCDs. However, a growing consensus has emerged that the risk of developing NCDs is cumulated throughout the life course, and thus screening in adults may be too late to reduce the risk and later interventions on adults may have limited effects [5].

## 3. Study of DOHAD: Observational Evidence

Numerous studies have investigated increased chronic diseases in later life and alterations in the development, structure and function of some tissues and vital organs. Both human and animal studies identified several parental factors such as diet, body composition, metabolism, changes in epigenetics, proteins and metabolites profile or microbiome can affect the perinatal outcomes [1,11,19]. Several observational human studies come from babies born following ART interventions that make this population (several million persons worldwide) one of the largest well-defined clinical cohorts [29]. In details, some of the ART procedures involve embryo culture and exposure to potentially inappropriate environmental factors, which may alter offspring phenotype [30]. The contribution of maternal and paternal influence represents a growing field of interest, promoting the study of female reproductive fitness and male fertility. 

Nevertheless, cellular models such as PSCs could also underpin research in DOHaD [31]. Indeed, the PSCs in vitro models allow studying the functional properties and developmental stages of tissues and support the principles of three Rs (3Rs: Replacement, Reduction and Refinement) of more ethical use of animals in testing by reducing the need for live animals. Cell-based models create space to manipulate pathways and mechanisms to consider additional downstream implications and assess therapeutic interventions. A summary of the models discussed below is shown in Table 1.

### 3.1. Human Studies

Investigating the influence of nutrition in utero has gained importance in the last three decades. The foetal cardiovascular system is highly susceptible to unbalanced nutrition during early gestation for various reasons. 

In the context of undernutrition, several pieces of early epidemiological data supporting the DOHaD concept come from well-documented famines and historical cohorts [32,33,34,35,37]. For example, during the Dutch Hunger Winter of 1944/45, analyses of maternal exposure to famine revealed that offspring had higher risks of developing cardiometabolic and neurological abnormalities, especially when their mothers were exposed to famine during the earliest stages of gestation [35,36]. Similarly, mothers exposed to the Chinese Great Famine (1959–61) during the first trimester of pregnancy gave birth to offspring who were four times more likely to develop hypertension in adulthood than individuals who had postnatal exposure to the famine [37].

Moreover, David Barker and his colleagues linked birth weight with the risks of cardiovascular diseases. Their studies used birth weight as an indirect factor of the intrauterine environment, their observations found an inverse relationship between the development of cardiac pathologies (high systolic blood pressure (SBP) and mortality from ischemic heart disease and birth weight [23,43]. Although initially the studies have focused on the effect of low birth weight and its association with heart diseases, in populations with a high prevalence of maternal obesity it has been shown that the risk for development of cardiovascular diseases has a “U” shape; this indicates that high birth weight individuals are also at risk of heart diseases [44,45].

On the other hand, high maternal Body Mass Index (BMI), obesity and increasing rates of type 2 diabetes mellitus (T2DM) during the PC period are also negative factors that could influence neonatal adiposity and the cardiometabolic profile in the offspring [38,40]. Undoubtedly, maternal overnutrition can be harmful to both mother and foetus, as a significant weight gain is associated with reduced fertility and decreased oocyte quality [42]. Moreover, maternal obesity might perturb the blastocyst glucose and insulin homeostasis, which might lead to an elevated foetal insulin and adult cardiometabolic dysfunction. In 2009, a study was conducted comparing the cardiometabolic risk between siblings with or without maternal obesity: siblings born before the mother underwent bariatric surgery (a set of surgical procedures used to treat obesity) had factors of metabolic risk significantly higher than siblings born after maternal weight loss [41]. The intrauterine environment is closely related to the development of diseases during pregnancy; during gametogenesis overnutrition leads to the accumulation of metabolites and inflammation in the mother’s ovarian follicles [39]. 

It is important to mention that the external environment to which a mother is exposed during the PC period or the pregnancy also influences the development of heart diseases. Epidemiological studies have linked that exposure to stress [80] or molecules such as nicotine can increase the risk of developing high blood pressure in the foetus later in life [81,82].

Despite the well-known link between a mother’s lifestyle and the long-term health of offspring, our knowledge of how the paternal factors contribute to the risk of adverse birth outcomes remains far less understood. Nevertheless, some recently published data linked adverse outcomes to paternal conditions such as sperm quality, epigenetic status and seminal fluid composition [1,2]. Male fertility is significantly affected by nutrition and body composition. As a matter of fact, an alteration from healthy BMI affects sperm motility, quantity and quality, and increases the damage from reactive oxygen species (ROS) [1,2]. Furthermore, overweight or obese men generally show a higher level of DNA damage in sperm than normal-weight males [47,51,53].

Epidemiological studies have shown that paternal exposure to various environmental factors can influence metabolic programming in offspring. This is the case with retrospective studies conducted on the population of Överkalix-Sweden and their problems with food supply due to crop failures in the 19th century. These data identified that people with an increased lifespan and lower risk of cardiovascular diseases had their paternal grandparents with poor access to food during their youth. However, grandchildren of paternal grandparents with excess food supply were more likely to suffer from diabetes and cardiometabolic diseases, correlating with a reduced lifespan, suggesting that paternal diet affects the offspring’s health [46,50,52]. Interestingly, the data from Överkalix-Sweden population contradict those of the Chinese famine mentioned above. This may be due to several reasons, such as differences between maternal and paternal inheritance, or different maladaptation responses to abrupt food uptake changes of the individuals. However, the exact mechanisms will need further investigation. 

Previously, it was thought that the primary information transmitted via sperm to the offspring was limited to genetic material. However, the role of the paternal germline now goes beyond this, as it is possible to transmit epigenetic information to the embryo, which plays an important role in the development and progression of health and disease [48,49]. Although it is not easy to study the direct relationship between epigenetic modifications in parental germline genes and the health of offspring in humans, epidemiological studies suggest a strong correlation [49].

Finally, as mentioned above, one of the most important sources of knowledge concerning developmental plasticity and DOHaD comes from babies born following ART interventions. Despite several millions of children conceived by ART and born healthy, little is known about the ART interventions’ long-term effects. Indeed, for the correct and safe application of human ART it is important to monitor the resultant offspring’s health status. Several human studies revealed different drawbacks of different ART techniques; for example, children could develop type 1 diabetes during childhood or poor cardiovascular health with increased risk of high blood pressure, vascular dysfunction and cardiovascular remodelling during development in utero [2,54,83]. Furthermore, ART-associated adverse effects on long-term health seem to have an epigenetic origin during the period around conception [84]. Indeed, a systematic review and meta-analysis of DNA methylation levels showed that IVF/ICSI-derived offspring have a higher likelihood of developing rare imprinting disorders compared to spontaneously conceived children [55]. 

The current challenge is to understand better the underlying mechanism of some factors affecting the embryo development and how to improve ART conditions to a level which eliminates adverse effects compared to in vivo conditions, with a special interest in the patient characteristics, hormone stimulation, laboratory procedures, culture media, oxygen tension during the embryo culture and cryopreservation [79]. 

### 3.2. Animal Models

Despite their physiological differences with humans, animals can model complications of human pregnancies; they are advantageous not only for developing and improving ART procedures, but also for investigating alterations that impact lifelong health. A wide range of species has been utilised [18,56,85], especially large animals (e.g., sheep, cows and pigs), which have numerous advantages and remarkable similarities to human beings [56,66]. Among them, the relatively long gestation period, the delivery of a single foetus in most cases with a fairly similar size to a human baby and numerous similarities in the function and structure of organs are examples of advantages of these models. For instance, several dietary models have been reported to use sheep in order to evaluate the effects of pre and periconceptional undernutrition on cardiovascular development [67,71]. Other studies have used the pig model to study the effects of a high-fat diet (HFD) during maternal gestation [68,72]. 

Moreover, the effects of a maternal HFD on the offspring have also been investigated in non-human primate models, showing that different body functions in the newborn can be negatively affected. For example, the offspring could have impaired glucose metabolism, liver dysfunctions and endothelial alterations [69,70]. Large animal models’ main disadvantages are the cost and the experimental duration, difficult genetic manipulation, together with numerous ethical concerns, especially regarding the non-human primate models [56]. For these reasons, the majority of DOHaD research has been conducted in rodent models such as rat and mouse due to their easy handling and housing, short gestation, low maintenance cost and the opportunity to perform genetic manipulations [17,56]. Likewise, rodent cardiac morphogenesis as well as adult cardiac structures are similar to those of human beings [57]. The early period of foetal development is sensitive in rodents, as several studies show blastocyst abnormalities and cardiovascular alterations in undernutrition or HFD models [58,59,65]. Nutritional deficiencies in rodents not only affect the foetal programming period but may also induce various pregnancy complications, negatively affecting the foetal environment and possibly triggering the development of CVD in offspring [60]. For example, stress, toxins or hypoxia cause the offspring to be small for gestational age, a risk factor for an increased incidence of CVD [63]. In addition, some studies in rodents have shown that the microbiome during development has a very important role in the occurrence of hypertension in the offspring. For this reason, as previously mentioned, exposure to a suitable environment during pregnancy can be key to improving long-term health and avoiding the development of CVD [61]. 

Paternal inheritance is also a critical aspect to consider in the development of CVD in murine models. Cardiovascular and metabolic health has been shown to be compromised in the offspring of obese fathers, and an epigenetic imprinting process in the sperm may cover a pivotal role [86,87]. These observations have been made in human studies, too [62,64].

### 3.3. Novel Approaches to Model the DOHaD Concept In Vitro: Focus on Cardiovascular Diseases

Studying developmental plasticity in the DOHaD concept and understanding the factors that can alter the offspring’s long-term health is challenging. CVD, like congenital heart defects (CHD), can result from genetic mutations or arise from malnutrition, drug-related effects, exogenous toxins, or maternal disease during gestation, leading to epigenetic changes [88]. 

Stem cells (SCs), as non-specialised cells, can give rise to more than 200 types of cells [73]. Over the past 20 years, stem cell biology has captured much attention in experimental research and cell therapy in order to counteract disorders such as neurological and cardiovascular diseases in both human and veterinary medicine [21,73,75,89]. Several types of SCs are used in the research field, but the PSCs, including ESCs and iPSCs, are currently the most attractive tool to study developmental biology and novel clinical applications [89]. Indeed, in the embryology field, PSCs have emerged as models to study the mechanisms that underlie embryonic development, covering the need to understand the development of tissues to predict and prevent numerous diseases caused by developmental defects [20,31]. The emergence of human embryonic stem cells (hESCs), derived from human blastocyst by Thomson and colleagues more than two decades ago [77], quickly made them an advantageous tool for human developmental studies. In 2007, the pioneer study of Yamanaka and his group generated human induced pluripotent stem cells (hiPSCs), avoiding the ethical concerns of hESCs and providing the opportunity to model patient-specific disease in a dish [78]. Human iPSCs are patient somatic cells that have been reprogrammed to acquire pluripotency capacity with unlimited self-renewal. The iPSCs are similar to the ESCs in gene expression and differentiation potential to give rise to any cell type of the organism. The hiPSC technology provides a unique in vitro platform to establish disease models, since the main advantage of using hiPSCs is that the cells possess the complete genetic background of the donor, including disease or risk-associated mutations [21,78]. Additionally, researchers can introduce and/or correct genetic variants with genome editing tools. This allows the generation of new disease models to determine personalised therapeutic strategies [21]. One of the powerful characteristics of this technology is that PSCs can spontaneously form aggregates named embryoid bodies (EBs), which are widely used as the initial step in several differentiation protocols, through exposure to specific differentiation signals [74,75,76]. 

In the DOHaD field, the usage of human PSC (hPSC) facilitates investigations of the factors that affect early human development [31]. In addition, hPSCs are a promising tool to identify potential biomarkers of epigenetic, cellular, metabolic and/or physiological changes during the early embryonic life and their effects on long-term health.

## 4. Epigenetic Background of Cardiovascular Diseases

Epigenetic status and heart development are strictly correlated [90], thus, comprehensive reviews have focused on the relationship between epigenetics and CVD risk factors, highlighting the importance of several biomarkers implicated in epigenetic mechanisms relating to [91,92]. 

Heart development is regulated by precise changes in gene expression, which are orchestrated by complex epigenetic mechanisms [93]. A mounting body of evidence from in vivo and in vitro studies implicates epigenetic modulation as a fundamental mechanism affecting the heart development and transmission of other alterations to future generations [58,94,95,96,97,98]. 

DNA methylation and histone modifications are highly dynamic in shaping the cardiomyocytes transcriptome during development and postnatal maturation [99]. The dynamic epigenetic status during the differentiation from progenitor cells to CMs has been investigated [100]; however, the detailed epigenetic process leading to cardiac alterations during embryonic development has been only recently investigated and not fully uncovered yet [99,101,102]. 

During cardiac development, DNA methylation and transcriptional changes have been identified for around 440 cardiac genes [99,101,103]. Of interest, methylation patterns of CMs exposed to pathological stress partially resemble those of foetal CMs, suggesting how the adaptation is firmly linked to changes in gene regulation and activity [97]. 

Histone modification has a central role in cardiac development, as well. Interestingly, pathological gene expression has been linked to changes of active histone marks, explaining up to 50% of pathological gene expression. For this reason, they can be considered as predictive markers for failing CMs [99]. In the biological process of cardiac diseases, histone demethylations are important epigenetic markers [104,105,106]. Specifically, JMJD2A histone demethylase, a member of the Jumonji protein family, seems to be correlated in the development of cardiac hypertrophy through the demethylation of H3K9me in a murine model [107]. It has been recently shown that JMJD2A can induce hypertrophy markers in the heart [108]. In a hiPSC-derived CMs (hiPSC-CMs) model, the JMJD2A effect seems linked to the demethylase activity in the ventricular (*NPPB*) and atrial (*NPPA*) natriuretic peptide regulatory region [108]. 

The intrauterine environment of mothers affected by diabetes, pre-eclampsia, obesity and intrauterine growth restriction have been attributed to long-term adverse effects, known as epigenetic priming of foetal development [109]. The heart responds to environmental signals by modifying the epigenome [58,110,111]; however, the details of the epigenetic status changes in CMs are still not fully explored.

A common response of the adult heart to various stresses is the re-activation of foetal cardiac genes by downregulation of adult transcripts, such as those involved in metabolism and calcium handling [112,113]. This response may also play a crucial role during early development. For example, enhancer of zeste homolog 2 (EZH2) represses the expression of *Six1* in cardiac progenitors to stabilise the postnatal cardiac gene expression [114]. Downregulation of *EZH2* in cardiac precursors destabilises cardiac gene expression and leads to pathological heart remodelling by the re-activation of foetal genes and pro-fibrosis factors. This causes postnatal myocardial pathology and upregulation of *Ink4a/b*, regulators of the cell cycle normally repressed by EZH2, leading to hypoplasia and decreased cardiomyocyte proliferation [114,115,116]. 

A significant part of the human genome is transcribed into noncoding RNAs (ncRNAs), which play crucial roles in CVDs and have been considered as potential biomarkers and therapeutic targets [115]. 

Of interest, dysregulation of numerous miRNAs has been shown if the foetus is exposed to a hostile intrauterine environment [95,96]. Neonatal heart development requires a broad regulation of numerous miRNAs, such as miR-15 and let-7 family members [117,118]. In the developing heart, aberrant overexpression of miR-195 (a miR-15 family member) results in heart abnormalities and premature cell cycle arrest [118]. On the other hand, knockdown of let-7 results in a significant decrease of hESC-derived CMs (hESC-CMs) size, area, sarcomere length, and expression of several maturation markers. These and multiple other miRNAs also play a role in disease progression [119]. For instance, knockdown of miRNA-133 leads to cardiac hypertrophy and re-activation of the foetal gene program in the adult mouse heart [120], while disruption of one of the two miR-1 family members, miR-1-2, has adverse consequences for development and maintenance of the heart, leading to ventricular septum defects [121]. 

Epigenetic modifications are involved in various biological processes, including foetal programming of normal development as well as disease predisposition. The studies reviewed here provided evidence that the epigenetic status of cardiac development is a highly complex mechanism involving miRNAs, DNA methylation and histone modification, with a crucial role in regulating CMs proliferation, maturation and remodelling. Of interest, this review will focus on CMs differentiation-related mechanisms and their potential transcriptional and phenotypic alterations following environmental toxicants. 

## 5. Modelling Cardio Myogenesis with Pluripotent Stem Cells

The heart is still immature at the end of organogenesis and during the whole perinatal period, while during the postnatal period it undergoes adaptations and metabolic switch to complete the maturation. Energy can be produced both from anaerobic glycolysis in the cytosol or through oxidative metabolism in mitochondria, and it is stored in the form of adenosine triphosphate (ATP) and phosphocreatine (PCR). The foetal heart is characterised by high glycolytic activity due to the low levels of circulating fatty acid and high lactate levels in utero, the major source of cardiac oxygen consumption [122]. During this period, glucose contributes to cardiac growth by hyperplasia. Indeed, glucose induces CMs proliferation in a dose-dependent way [123]. Immediately after birth, to promote the metabolic switch to fatty acid oxidation, glucose uptake is drastically reduced in the foetus, and this phenomenon is also accentuated by the first meals of the newborn from maternal milk [122]. As cardiac energy demands increase immediately after birth, the mitochondria count grows considerably in the CMs, as well as their mass, due to the activation of transcription factors and regulators [124]. Among them, the nuclear receptor factors peroxisome proliferator-activated receptor (PPARs) and their coactivator like the PGC-1α are important for the mitochondrial biogenesis, as well as nuclear erythroid 2-like 2 (*NFE2L2* or NRF2) that provides the transcription of antioxidants in order to balance the high ROS production from the mitochondrial oxidative phosphorylation [122,124]. 

**Figure 2:**
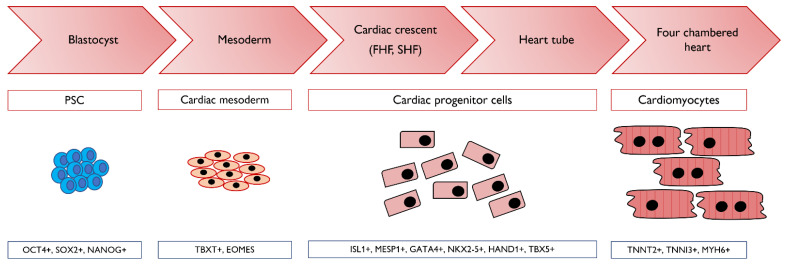
Comparison of cardiac development during prenatal days with PSC differentiation stages. Key genes are also shown at each time-point. FHF: First Heart Field, SHF: Second Heart Field.

One of the advantages of PSC technology is the in vitro generation of cardiac tissue following precisely the embryonic differentiation and development of the tissue, as shown in Figure 2 [125,126]. 

This tool allows the investigation of differentiation and maturation processes of CMs, also of changes, interactions and possible alterations that can affect cardiomyogenesis in the early stage of gestation. Human CMs can now be easily produced in large quantities from human PSC and used for the study of cardiac physiology and pathophysiology [127,128]. Many cardiac tissue PSC models are available to date. For example, studies have reprogrammed and differentiated adult somatic cells into pluripotent stem cells to generate CMs in vitro [129,130]. Especially, the main goal is to reproduce in vitro the in vivo cardiac architecture as well as possible. In a 2D model, this can be achieved by differentiating cardiac cell populations, such as CMs, endothelial cells and vascular mural cells, collecting and re-plating them to construct cardiac tissue sheets [130]. Since in 2D cultures it is arduous to fully recapitulate the heart’s unique cytoarchitectural arrangement, to bring this tool to an even higher level of similarity to the original tissue, it is necessary to develop a 3D cellular model, for generating CMs spheroids as well, using different methods, such as suspension bioreactors and the formation of EBs [129]. Indeed, a functional heart reconstruction requires not only a resource of heart cells, but also a complex tissue arrangement, including matrices and vascular structures. To combine tissue engineering, stem cell biology and heart developmental biology into a new platform, there are studies on repopulating decellularised rodent hearts with human ESC or iPSC. This might be a novel strategy to generate artificial humanised hearts, even if their pumping capacity is still very low compared to normal hearts [131,132]. However, CMs spheroids are still the most used 3D model due to their easier handling and observation, as well as lower costs compared to a whole 3D heart. 

Despite the advantages of hiPSC-CMs in 2D and 3D cultures, the main challenge is still to obtain functional and mature CMs in terms of contractile structure, metabolism and electrophysiological properties. For these reasons, many research groups have focused their studies on developing an efficient and cost-effective protocol that provides a well-established in vitro model that could recapitulate more faithfully the in vivo physiology. Available protocols for hPSC cardiac differentiation require the accurate regulated expression of multiple families of secreted growth factors, such as transforming growth factor-β (TGF-β) superfamily members, like activin A, BMP2 and/or BMP4 and modulators of canonical Wnt signalling [125,133,134]. At the same time, it is relevant to focus on the long-term culture of CMs to increase their maturation. Seeing as hPSC-CMs correspond to the foetal state for their functional and physiological characteristics [135,136], the study of long-term alterations in adulthood can be challenging to analyse. Available examples in the literature provide interesting strategies to promote CMs maturation, such as repressing hypoxia-inducible factor α (*HIF1α*) [137], overexpressing the miRNA let-7 family [117], or supplementing the culture medium with fatty acids [138]. However, more complex approaches are also under evaluation. Especially, the maturation of CMs can be improved by controlling the surrounding extracellular matrix [139], applying mechanical and electrical stimulation [140], as well as using dynamic culture [129,141]. Ruan and his group showed that mechanical stimulation improved the sarcomere alignment and formed stiffer constructs, while electrical stimuli improved contractility [140]. Of interest, dynamic cultures overcome the limitation of 2D models since they allow the production of large numbers of hiPSC-CMs and increase the functional genes and contractile proteins expression of CMs [129,141]. In this regard, it is necessary to consider the influence of shear stress that could alter cell viability and proliferation through physical damage and cell death. Despite this drawback, the appropriate application of shear stress is an effective approach to improve cardiac differentiation efficiency and maturity [129,141]. 

Furthermore, PSC-CMs are a valid model to study the epigenetic status during cardiac programming of disease state as well as normal development [100]. Recent evidence shows how hiPSC and hESC-CMs are used to investigate the functional role of structural epigenetic changes in the heart, such as the 3D chromatin topology dynamics, during development and disease [142,143,144]. Indeed, it is known that alterations in genome topology play key roles in CHD and CVD and can represent promising targets for therapeutic intervention [145,146]. Moreover, by using PSC-CMs is possible to control the in vitro environment, and thus specifically test for genetic or epigenetic effects in response to controlled perturbation [147]. 

Summing up, these approaches will improve many facets of cardiomyocyte maturation but most important they will give a model to evaluate the long-term effect of several stressors affecting the health of cardiac tissue during its development.

## 6. Studying the Effects of Environmental Factors on Cardiomyocytes Differentiation

As mentioned, aside from genetic mutations, several environmental factors (e.g., diet, oxidative stress, chemicals or pathogens), maternal diseases or other causes like ART procedures (e.g., regiments for the stimulation of ovulation followed by IVF such as ICSI) can lead to cardiac diseases in the newborn. Congenital heart disease is the most common cause of neonatal mortality related to congenital disabilities. Although genetic factors play a significant role in CHD development, a genetic diagnosis is established for only 11% of the individuals [148], highlighting the crucial role of non-genetic contributors. Moreover, many other complications can result from both environmental and nutritional stressors without any genetic predisposition. For example, ischemia-reperfusion injury, metabolic dysregulations and hypertension lead to heart failure in adulthood [149]. Pluripotent stem cells derived CMs can be exposed in vitro to various stressors to investigate their effects on CM function.

### 6.1. Nutritional Effects on Cardiomyocytes Function

Maternal HFD or elevated maternal glucose and insulin concentrations are adverse effects of high maternal BMI, diabetes and obesity, which can affect the cardiometabolic functionality in offspring, as previously mentioned. Similarly, lipid and fatty acid accumulation are strictly correlated with CVD predisposition [1,2]. Maternal HFD has also been shown to reprogram cardiac metabolism and induces cardiac hypertrophy [150,151] and myocardial cell fat deposition [152]. Interestingly, Watkins et al. found in a murine model in which mothers had a low protein diet during the pre-implantation period (days 0 to 3.5 of embryonic development), that the offspring showed arterial hypertension during postnatal life [153], as do offspring exposed to maternal low protein diet during the whole pregnancy [154,155]. This was confirmed in vitro with a culture of mouse embryos in different concentrations of insulin and branched-chain amino acids (known as factors that induce the programming of a low protein diet), showing an increase in SBP in the offspring [156]. Moreover, maternal undernutrition reduces offspring CMs [157] by increasing apoptosis [158] and/or reducing proliferation [154,159].

A metabolic condition strictly related to nutrition and widely studied is maternal diabetes before or during pregnancy (also known as gestational diabetes), which is associated with increased early-onset CVD rates [160]. Diabetes in mothers is characterised by poor maternal glucose control, leading to the heart remodelling or defects in the foetus that predispose to risks of cardiac complications in adulthood [123,161]. In gestational diabetes mellitus (GDM), glucose homeostasis can be affected by increased maternal levels of oestrogen, progesterone, cortisol and human placental lactogen [160]. In all cases, pregnancy complicated by diabetes involves large amounts of maternal glucose freely cross the placenta, leading to increased secretion of foetal insulin [162]. This increase exposes the foetus to hyperinsulinemia and hyperglycemia with long-lasting effects on the embryonic heart and foetal vascular gene expression, resulting in vascular function changes and contributing to higher CVD risks of hypertrophy [160]. To further complicate the picture, exogenous insulin therapy in diabetics can lead to hypoglycemia as a common side effect. Thus, maternal hypoglycemia has been linked with the disorganisation of myocardial layers, cardiomegaly and heart failure in several in vivo and in vitro animal studies clearly collected in a review of Ida W. Smoak [163]. However, the molecular mechanisms by which maternal diabetes may cause the risk of developing CVD in offspring is still poorly understood [160,162,163]. 

Both human ESC and iPSC are used for studying the effect of impaired glucose homeostasis on the cardiac lineage [123,161,164], as well as the effect of nutritional overconsumptions [165,166]. As described by Nakano and colleagues, if glucose uptake is not reduced in foetus just before birth, the nucleotide biosynthesis supported by high levels of glucose can inhibit cardiac maturation [123]. As a consequence, the delay of the metabolic switch may lead to pathological remodelling of the neonatal heart, contributing to the onset of CHD. The impact of glucose exposure on cardiac differentiation was analysed using hESC-CMs cultured in media containing various glucose concentrations, which dose-dependently suppressed the expression of key cardiac markers *TNNT2* and *NKX2-5*, as well as the mitochondrial marker *PPARGC1A* [123]. Similarly, Balistreri and his group aimed to assess the effects of high glucose on foetal cardiac development, generating hiPSC-CMs in microtissues within silicone micro molds. They showed that elevated glucose exposure impaired the ability of CMs to self-assemble into the 3D model cardiac tissue, as well as CMs calcium handling function [161]. Moreover, hiPSC-CMs exposed to prolonged hyperglycemia show pathological hypertrophy and reduced contractility due to calcium cycling dysfunctions [164]. Following nutritional alterations, phenotype as cardiac hypertrophy, disorganisation of myocardial layers and alteration of contractility rate has been observed in animal models, too [150,151,152].

Modelling cardiac non-genetic conditions allows for studying the effects of nutritional stressors. For example, approaches of metabolic overload with fatty acids can recapitulate insulin resistance condition [165,166]. Overconsumption of lipids is correlated with high risks of developing heart failure. In this context, CM accumulate elevated rates of long-chain fatty acid (LCFA). Therefore, the oxidation capacity of the mitochondria is overloaded, inducing mitochondrial dysfunction over time. Upon high palmitate culturing, hiPSC-CMs developed the main features of insulin resistance such as loss of insulin-stimulated LCFA/glucose uptake and increased basal LCFA uptake [166]. Ultimately, lipid oversupply leads to CMs contractile dysfunction. The human pathological model of insulin resistance can be recapitulated in hESC-CMs with either TNFα or free fatty acids (FFA), both leading to higher transcription of proinflammatory markers *NFKB1*, *IL6* and *CXCL8* and the inhibition of *PPARGC1A* gene expression [165].

### 6.2. The Role of Hypoxia and Reactive Oxygen Species on Cardiomyocytes

Stem cells in vivo occupy a hypoxic niche, and their energy metabolism is mainly dependent on glycolysis for ATP generation [167]. However, the relationship between hypoxia and differentiation of stem cells is a matter of debate yet. Hypoxia alone can revert committed cells back to an undifferentiated-like state, together with other reprogramming factors included in the process [168]. At 2% of oxygen concentration, hypoxia-inducible factors (HIFs) are stabilised in the niche, working as a de-differentiation rheostat. However, the detailed mechanism under this process is still under investigation [168]. By contrast, hypoxia seems to play an important role in the proliferation, differentiation and maintenance of committed cells, among them CMs during development. In this context, the exogenous expression of *HIF-1α* has been shown to promote cardio myogenesis in ESC [169]. To further complicate the picture, it is widely known that exposure to hypoxia during foetal development has the potential to cause abnormal heart morphology and function [170,171]. Additionally, hypoxia has been shown to impair foetal CMs proliferation, followed by an increase of apoptotic events [172]. Medley and colleagues employed a mouse iPSC-CMs (miPSC-CMs) based approach to investigate the mechanism by which hypoxia influences cardiomyocyte development. In this work, miPSC-CMs exposed to relatively short-term hypoxia exhibit a long-term failure to develop of a contractile phenotype [173]. Furthermore, in Gaber et al. [174], hESC-CMs were exposed to 1% hypoxia for 72 h, which was followed by an increase of *HIF1α* expression. They recapitulated the foetal hypoplastic left heart syndrome (HLHS) in a chronic hypoxia model. Consequently, hESC-CMs displayed more DNA damage, transcript alterations and senescence with reduced cell proliferation and fewer cardiac progenitors [174]. Interestingly, Kobayashi et al. supported transcript alterations in patient HLHS-derived CMs, linked to epigenetic modification of important cardiac genes involved in the early cardiac development program, as *NKX2-5*, *HAND1* and *NOTCH1* [175]. Of particular note, ChIP assay suggested that reduced H3K4me2 and increased H3K27me3 on the *NKX2-5* promoter might be the epigenetic mechanism that leads to impaired transcriptional expression in the differentiation processes of HLHS-derived iPS cells, thus causing critical defects for cardiac differentiation and heart morphogenesis [175]. Following hypoxia exposure, epigenetic and transcriptional alterations have been observed in animal models, too [176,177].

Hypoxia, as well as other factors like environmental pollutants [178,179] and hyperglycemia [164,180], are known to trigger the production of reactive oxygen species (ROS), altering cellular oxidative homeostasis [181,182]. ROS are derived from molecular oxygen and are produced in subcellular compartments as highly active molecules, ions or radical (e.g., hydrogen peroxide, superoxide and hydroxide) with a pivotal role in cellular metabolic activity, particularly during cardiac differentiation and development [183]. Their physiological or pathological effects depend on their spatiotemporal source, such as the release duration, the local levels and interaction with different subcellular compartments [183,184]. NADPH oxidases, the mitochondrial electron transfer chain and uncoupled NO synthases are important ROS sources [183]. Superoxide anions can be converted to hydrogen peroxide (H_2_O_2_) via superoxide dismutase (SOD), and they can also react via different pathways (e.g., Fenton reaction) to generate hydroxide anion [183]. High ROS levels activate the nuclear factor erythroid 2-like 2 (*NFE2L2* or NRF2), which regulates the production of antioxidant defences (e.g., glutathione peroxidase, thioredoxins, heme oxygenase-1, NAD(P)H dehydrogenase 1) [185]. 

The main source of hypoxia-induced ROS comes from the complex I and III of the mitochondria electron transport system [182]. Under normal conditions, electrons flow freely in the electron transport system, reducing the time at which free radicals can interact with molecular oxygen. During hypoxia, the flow rate of electrons slows and the molecular oxygen can gain an unpaired electron to produce superoxide [182]. Since hypoxia can alter redox status, significant changes in cellular homeostasis and subsequent changes in gene expression can occur. It is still unclear what signals mediate repression of cardioprotective genes in utero during hypoxia, however, stress factors such as the trigger of oxidative stress may be involved [171]. Under hypoxic conditions, CMs are subjected to increased oxidative stress [186]. The molecular mechanism of prenatal hypoxia impacting foetal heart development and the risk of heart disease in adulthood is still poorly understood. Recently, Chen et al. showed that prenatal hypoxia induced a global epigenomic reprogramming, strictly linked to long-lasting effects on the adult heart [176]. Indeed, previous studies showed that hypoxia is involved in in utero epigenetic programming, leading to an inhibition of cardioprotective genes (e.g., *PKCε*, *HSP70* and *eNOS*) in foetal CMs [111,177,187]. 

**Figure 3:**
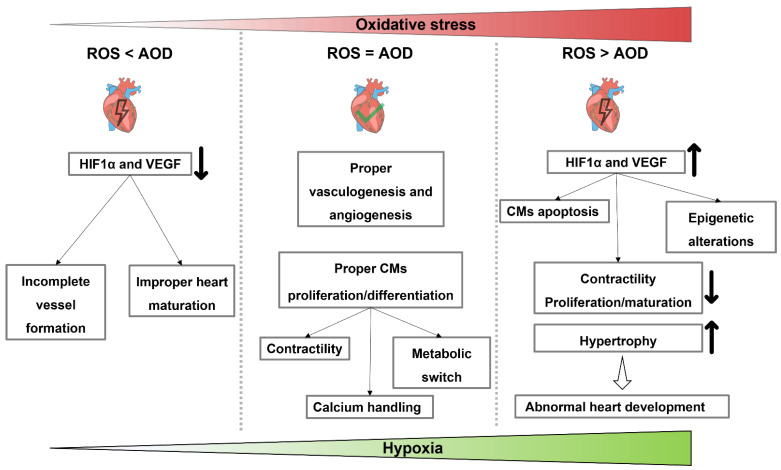
Unbalanced exposure to hypoxia and ROS impairs the antioxidant defences (AOD) and causes alterations to foetal CMs. The affected regulation of cardiac-specific genes, epigenetic modifications and the aberrant cardiac remodelling can lead to CVD in the newborn. AOD: antioxidant defences.

In the human body, ROS play a contradictory role. The limit between a beneficial and deleterious response (oxidative eustress and distress, respectively) remains to be clearly evaluated in health and disease [183]. Evidence shows that normal levels of ROS play a critical role in cellular homeostasis and function during the cardiovascular commitment [188,189,190]. Indeed, Li and colleagues showed a link between H_2_O_2_ treatment and the gene expression of cardiogenesis, demonstrating that ROS signals are indispensable in modifying cell fates through the induction of cardiac-specific genes such as *GATA4*, *NKX2-5* and *MEF2C* [184]. On the other hand, H_2_O_2_ stress stimuli are highly dependent on the duration and the magnitude but also on the differentiation status of the cells. Indeed, they showed that short-time stress of H_2_O_2_ could promote cardiac commitment, but the excessive ROS stress damages the cardiomyocyte’s differentiation and contractile phenotype [184]. The production of ROS is essential to induce the maturation of CMs, and in vitro studies confirmed this hypothesis [190]. Specifically, ROS are associated with the improvement of CM functions such as contractility [191], calcium handling [192], metabolic switch [193] and hypertrophic growth [194]. However, overproduction of ROS can lead to cellular oxidative stress, resulting in abnormal embryogenesis [195,196,197] (Figure 3).

As mentioned above, high ROS levels trigger antioxidant defences and, among them, NRF2/KEAP1 and endothelial NO synthase (eNOS) pathways are probably the most relevant in the cardiac system [198]. *NRF2* is a transcription factor that binds antioxidant response element (ARE) to regulate the production of antioxidant enzymes. NRF2 is abundant in all tissues, but in the heart it counteracts conditions such as hypertrophy, myocardial infarction, atherosclerosis and hypertension [185,199]. *NRF2* is negatively regulated by Kelch-like ECH-associated protein 1 (KEAP1), which, under unstressed conditions, mediates the NRF2 turnover and ubiquitylation in the cytoplasm [185]. In response to oxidative stress, KEAP1 is oxidised and inactivated, resulting in NRF2 stabilisation and translocation into the nucleus [185]. *NRF2* is highly important in the cardiac system [199] and plays a pivotal role during pregnancy, protecting the foetus from adverse oxidative stress conditions in utero both during early and late development [109]. On the other hand, nitric oxide (NO) is another important regulator of the cardiovascular system during its development [200]. The NO is a vasodilator produced from the eNOS and its role is crucial in preventing the pathogenesis and progression of heart diseases [200]. Indeed, the downregulated protein expression or the uncoupled activity of eNOS predispose to heart alterations [200,201]. NRF2/ARE signalling is highly correlated with eNOS pathway through the PI3K/Akt activation [198]. Under redox imbalance, PI3K/Akt pathway activates *NRF2* and its downstream targets, and on the other side regulates *eNOS* activation and NO production, which targets NRF2 signalling again. Pathophysiological conditions in heart such as fibrosis, apoptosis, hypoxia, ischemia trigger PI3K/Akt and other pathways to activate NO-NRF2/ARE axis to counteract cardiac and vascular complications [198]. 

Oxidative stress has been suggested to alter the epigenetic status of the heart [202]. Additionally, in the ART area, in vitro manipulation of gametes or embryos makes it difficult to maintain pro and antioxidant balance, and the increased ROS levels are one of the major triggers of early life epigenetic changes with long-lasting effects in adult life [203]. Of interest, among several epigenetic changes, the antioxidant systems NRF2/KEAP1 and eNOS have been proposed as targets of epigenetic priming during foetus development under adverse intrauterine environment [109,204]. For instance, West and colleagues have listed several methylation patterns in the offspring of diabetic mothers. In particular, they found hypermethylation of DJ-1 (*PARK7*), a stabiliser of NRF2. This epigenetic variance was previously linked to vascular endothelial cells’ alterations as it potentially decreases the NRF2 protection in the vasculature [205]. Furthermore, in conditions such as diabetic cardiomyopathy, *NRF2* alterations have been correlated with *KEAP1* promoter demethylation that leads to the transcription factor ubiquitination [206]. In another work, Sherrer’s team found that increased methylation on the promoter of the *eNOS* gene led to a reduction of NO levels in plasma. This increased blood pressure and vascular dysfunction and caused a shorter lifespan in mice born following ART procedures in mothers exposed to a high-fat diet [207]. Even if epigenetic changes occurring during high glucose stress or hypoxia are still under evaluation, evidence links them to *eNOS* dysfunction. For example, maternal hyperglycemia could affect the eNOS activity by reducing the chromatin accessibility at the *NOS3* locus [208]. On the other hand, hypoxia causes a significant decrease in H3/H4 acetylation of *eNOS* proximal promoter histones [187]. 

Human PSC can be a novel in vitro model to study the effects of oxidative stress on the early embryo [209,210], as suggested by several studies mentioned in this section, as well as to investigate the changes in the epigenetic status of CMs under hypoxia and oxidative stress. 

### 6.3. Alcohol Consumption and Cigarette Smoking

Maternal alcohol consumption and cigarette smoking leading to exposure during the gestational period can affect the foetus development and cause heart alterations, with negative implication for postnatal cardiac function [178,211,212,213]. However, there are few in vitro studies concerning the effects of these substances on CMs differentiation and maturation yet. Concerning the effect of maternal alcohol consumption, a clear and recent example is provided by Rampoldi et al. who evaluated the impact of ethanol on hiPSC-CM functionality as a model of prenatal exposure during maternal alcohol intoxication [195]. Ethanol exposure of hiPSC-CMs results in reduced cell viability, increased cell loss, and ultimately leads to overproduction of ROS. This study elucidated that, despite the activity of key calcium handling proteins being modulated by ROS, as RyR2 (cardiac ryanodine receptor) and SERCA (SR calcium transport ATPase) [214], the impaired levels of these products contribute to abnormal calcium handling. Indeed, treatment of hiPSC-CMs with the ROS scavenger N-acetyl cysteine reduced the ethanol-induced ROS production and abnormal calcium transients in hiPSC-CMs [195]. Interestingly, RNA-seq detected significantly altered genes, among which members of the potassium voltage-gated channel family and solute carrier family [195]. Furthermore, ethanol has been reported to have toxicity on mouse ESC-CMs (mESC-CMs), inducing mESC growth inhibition and the delay of cardiac differentiation through the Wnt/β-catenin signalling pathway suppression [215]. On day 11 post-differentiation, ethanol significantly suppressed the expressions of important cardiac transcripts required for the differentiation (i.e., *NKX2-5*, *MEF2C*, *TBX5*, *HAND2* and *aMHC*) and maturation (i.e., *CX43* and *TNNC1*). Transcriptional alterations of specific cardiac genes and increased oxidative stress have been also observed in foetal mice, leading to heart dysplasia and CHD [216]. Indeed, alcohol exposure reduces histone methyltransferase (HMT) activity in the heart [216]. Among different HMTs, G9α-HMT is closely related to cardiac development [115,216]. Under alcohol exposure, the suppressed activity of G9α-HMT seems linked to H3K9me3 hypomethylation, which in turn promotes the overexpression of cardiomyogenesis-related genes (e.g., *MEF2C* and *CX43*). This mechanism may be involved in alcohol-induced cardiac dysplasia, leading to CHD in foetuses [216].

Detrimental effects are also experienced with foetal exposure to maternal cigarette smoking due to many toxic chemicals such as formaldehyde, benzene, toluene, phenols, nicotine, etc. [178,213]. According to a population-based study, it is well known that smoking mothers have a high risk of affecting heart development and function in the offspring [217]. In this regard, Cheng et al. conducted a study concerning the effect of cigarette smoke on cardiac development in vitro [218]. Exposure of cigarette smoke to mESC-CMs impairs cardiac-specific genes expression (e.g., *GATA4*, *NKX2-5*, *MEF2C*, *α-MHC* and *MLC1a*) through the BMP-SMAD4 signalling pathway. Despite cigarette smoke inducing apoptosis in mESC-CMs, those cells that survive can undergo further differentiation, potentially risking abnormal heart development, leading to CHD [218]. Notably, the non-cytotoxic dosages of cigarette smoke significantly decreased global histone H3 acetylation level in mESC-CMs. In particular, low levels of histone acetylation were observed in the promoter regions of *GATA4*, *MEF2C* and *NKX2-5* [218]. Histone acetylation promotes the relaxation of chromatin structure for the transcriptional activation and, during CMs differentiation, it controls transcription of several cardiac genes, like *GATA4*, *MEF2C* and *NKX2-5* [219]. Thus, alterations in this epigenetic regulation contribute to CHD [220]. Furthermore, a recent study conducted by Guo et al. [212] shows that 6-day exposure to nicotine reduces the viability of hESCs, increases ROS and alters cell cycling in hESC-derived EBs, suggesting that nicotine affects embryo development as early as the pre-implantation stage. In addition, Ca2+ signalling was found to be affected in hESC-CMs upon nicotine exposure, increasing the propensity to Ca2+-associated arrhythmia [212]. Electronic and conventional cigarette smoking extract impaired hiPSC-CMs function, slowed beating and increased ROS-induced cell death [221]. Notably, RNA-seq revealed numerous altered genes essential for normal heart function and response to stress, including *MYLK*, *NPPA*, *TNNT2* and *TNNI3*. Most of them were downregulated, probably due to a significant increase in upstream methylation signals (for both DNMT3A and B pathways) [221]. Interestingly, pathological heart remodelling, transcriptional and epigenetic changes have been also observed in mice [213]. 

Overall, the studies revised in this paragraph show that using iPSCs-derived CMs will facilitate to investigate cellular toxicities and transcriptional profile changes triggered by alcohol and cigarette smoking, affecting CMs functionality.

### 6.4. Glucocorticoids

Many of the hormones produced by the placenta are essential for proper foetus growth [222]. In this context, the hypothalamic–pituitary–adrenal axis plays a pivotal role during embryonic development with potent programming effects on organ development [222]. Recent studies support the role of glucocorticoids in regulating CM development [223,224,225,226]. In vitro studies report that thyroid and glucocorticoid hormones are critical for CMs maturation, also suggesting a method to improve PSC-CMs differentiation efficiency and maturity [224,225]. However, future assessment of the effects of these hormones in vitro is needed. 

Maternal diet and other stressors may modulate hormonal secretion patterns and alter the uterine environment, compromising the integrity of the placenta itself, the gestation length and the foetal growth rates [222,227,228]. Moreover, despite that glucocorticoids are able to improve neonatal survival in preterm infants, an excess of exogenous glucocorticoids during pregnancy is related to reduced birth weight and adverse outcomes in the offspring, especially if glucocorticoids are administered during late gestation when growth is speeding up, and it is probably most susceptible to the catabolic effects of steroids [227]. The excess of glucocorticoids exposure during pregnancy increases the myocardium susceptibility of male offspring’s heart to postnatal injury due to the decrease of protective factor BMP4 caused by the hypermethylation on *BMP4* promoter, in all likelihood correlated to mitochondrial damage and myocardial susceptibility to injury [229]. Despite glucocorticoids being necessary for cardiac maturation, excessive in utero exposure to glucocorticoids can negatively impact the maturation process. Indeed, a high concentration of maternal cortisol throughout late gestation alters the regular cardiac gene expression pattern in the ovine foetus [230,231], which persists into postnatal development. Transcriptomic profile of lipid metabolism, cell proliferation and cardiac remodelling, are affected postnatally after the in utero cortisol exposure, together with increased cardiac wall thickness and altered glucose metabolism [232]. These alterations may cause postnatal cardiac hypertrophy and altered responses to oxidative stress [232]. In addition to higher cortisol release, stress triggers the increase of norepinephrine and inflammation, which affect the foetal environment and lead to infant health complications. This may lead to conotruncal heart defect and neural tube defects in offspring, and the risk of delivering a low-birth-weight infant as well as preterm birth [233].

### 6.5. Chemical Exposure

Exposure to environmental pollutants may cause severe toxicity problems, resulting in infertility, early spontaneous abortion, developmental defects or cancer [178]. Pesticides, antibiotics and industrial excipients are chemical substances widely used in agriculture, medicine, and the chemical industry. It is well known that they can circulate from the maternal blood into the developing embryo or foetus via the placenta, causing developmental toxicity as well as malfunction of organs after birth, such as the heart [178]. A low concentration of flusilazole, a pesticide agent, can inhibit the differentiation of mESCs into CMs. In addition, this substance decreases the viability of mESCs by about 50% and reduces cardiac differentiation rate in a dose-dependent manner, leading to a significant change of cardiac differentiation-related gene expression [234,235]. On the other hand, some antibiotics are known to cause cardiac disorders like cardiac arrhythmias. A clear example is provided by sparfloxacin and levofloxacin that were shown to markedly change the frequency and rate of beating in mESC-CMs [236]. Furthermore, industrial excipients play a critical role in developmental toxicity [178]. For example, trichloroethylene hinders the CMs maturation and the Ca2+ dependent contractibility in hESC-CMs, while perfluorooctane sulfonate alters the expression of cardiac-specific genes and can induce mitochondrial damage in mESC-CMs [237,238]. Moreover, bisphenol A (BPA) is an organic compound used in the production of various materials like plastics and, due to its accumulation in human tissues and organs, it is potentially harmful to human health [239]. Exposures to BPA on mESC-CMs affected the morphology of the cells, enlarging the cardiomyocyte size, increasing collagen expression and damaging the mitochondria [240]. On the other hand, hESC-CMs exposed to non-cytotoxic BPA concentrations showed higher expression of hypertrophic-related transcript levels (such as *NPPA* and *NPPB*), increased cellular size and reduced ATP provision due to changes in mitochondria features [241]. Due to its hormone-like properties, BPA may bind to oestrogen receptors [239,242]. Notably, BPA embryonic exposure seems to affect the cardiac phenotype through the oestrogenic and epigenetic pathways, increasing the expression of the oestrogen receptor (*ESR2b*) and promoting the over-expression of a histone acetyltransferase (*KAT6a*), which causes an increase in histone acetylation. Both mechanisms might act in synergy and can lead to the upregulation of *HAND2*, a crucial factor for CMs differentiation [242]. The overexpression of *HAND2* has been correlated previously with excessive proliferation of cardiac progenitor cells, leading to malformations in the heart tube and the ventricular outflow tract [243]. A summary of the models revised above is shown in Table 2.

## 7. Concluding Remarks

Here we summarised the main features of the DOHaD concept, highlighting that several stress factors during foetal and perinatal life can influence the future health of the individual and increase susceptibility to adult diseases, such as CVD. The epigenetic state can be modified by maternal environmental influences, such as high glucose, oxidative stress, hypoxia, which in turn alter DNA methylation and modify histones [7]. Moreover, a wide variety of environmental toxicants including cigarette, alcohol, chemicals and hormones have a role in epigenetic aberrations [7,213]. Since there is an obvious connection between altered conditions during the PC period and the risk for the offspring to develop CVD in adulthood, studying alterations during early cardiac development might allow efficacious disease prediction and prevention for future generations. In such case, the use of PSC-CM becomes essential to study the onset of CVD, given their incidence later in adult life. 

The advent of PSC technology has permitted assessing the tissue functional properties and studying the stages of tissue development, enabling to recapitulate organ-like complexity and functionality. PSCs may be a useful tool to investigate early developmental toxicities of various stress due to their pluripotency that recapitulates the dynamic nature of embryonic development. Recently, PSC-CMs have also become an important in vitro model for toxicity screening [212,244]. This tool may help to understand better the degree of individual, genetic susceptibility to stressor-induced cardiotoxicity. However, stress exposure in vitro, for practical reasons, is different from the in vivo stress exposures occurring over a longer period of time and in variable doses. Developing a more sophisticated in vitro model system with longer differentiation time might help to overcome the current limitations. Moreover, comparing the exposure effects in variable specific time points of the in vitro cardiac differentiation and extrapolating to in vivo development might allow to generate data comparable to the stress exposures occurring in vivo. Detecting the most sensitive stages of differentiation in vitro would be helpful to identify such in vivo stages, too. In addition, the PSC-CMs models discussed in this review showed that stress exposure leads to epigenetic alterations, such as dysregulation of genes involved in CMs differentiation or functionality, which are studied to investigate the role of structural epigenetic changes in the heart during early development. Even if PSCs can be an effective model to reduce the use of experimental animals, which is costly and time-consuming, and involves numerous ethical issues, using PSCs instead of animals is still a controversial issue because it is difficult to predict the in vivo results with only in vitro data [66]. Moreover, even if the features of PSC-CMs are improving due to numerous efforts [245], differentiation methods still need further improvement to reach the desired degree of maturity. Indeed, this represents a major difficulty to recapitulate the adult phenotype and, therefore, an adult disease modelling observed in vivo. On the other hand, the maturation of ESC/iPSC-derived CMs from embryonic stage represents a unique opportunity to evaluate the disease progression from early stages of development to the adult tissue and to understand late-onset changes, as well. 

In the near future, it will be essential to efficiently identify determinants of NCDs during the entire life course, including the PC period that is a (perhaps the most) critically important period in which it might be possible to intervene to improve human health during the rest of the life course. The most challenging part will be to define the epigenetic basis of DOHaD and whether epigenetic environmental changes associated with CVD risks are heritable. The reprogramming process from patient somatic cells to iPSC is removing the majority of epigenetic marks. The experimental reestablishment of epigenetic markers during differentiation might offer insights on understanding the role of genetic background in individual responses to environmental stressors contributing to DOHaD. Subsequently, it will be relevant to propose appropriate interventions to reduce an individual’s risk of developing these conditions.

## Figures and Tables

**Table 1:** Advantages and disadvantages of the models to study DOHaD and CVD risks.

	Human Studies	Animal Studies		hPSC Models
		Small Animals (Rodents)	Large Animals	
PROS	Data supporting DOHAD: Undernutrition [32,33,34,35,36,37] Overnutrition [38,39,40,41,42] Birth weight [23,43,44,45] Paternal contribution [1,2,46,47,48,49,50,51,52,53] ART technique [2,54,55]	Easier handling/housing and genetic manipulation [17,56] High sequence conservation with humans [57] Data supporting nutrition and pregnancy complications [58,59,60,61,62,63,64,65]	Similarities with human [56,66] Data supporting nutrition and pregnancy complications [67,68,69,70,71,72]	Unlimited supply of genetically well-defined material [73] Recapitulate embryonic development [31,74,75,76,77] Possess the complete genetic background of donor/patient [21,78] Easier to introduce and/or correct genetic variants [21] Disease modelling in human cells/tissues [21,78]
CONS	Necessity of long-term data, larger prospective cohorts and expensive longitudinal studies [2,79]	Physiological differences with humans [56,66] Genetic manipulation not always reflect the pathogenic mutation in human [57]	Cost and experimental duration [56] Difficult genetic manipulation [56] Ethical concerns [56,66]	Difficulty to predict in vivo readouts with only in vitro data [66] Difficulty to resemble the native tissue/multi-organ complex environment [66] Genetic instability [21] Phenotypic heterogeneity between iPSC lines [21] Incomplete maturation of iPSC-derived cells [21]

**Table 2:** Effect of several stressors on pluripotent stem cell-derived cardiomyocytes.

Model	Condition	Stimulus	Key Phenotype	Reference
hESC-CMs	Hyperglycemia	High-glucose exposure	➢ CM maturation inhibition ➢ Suppression of *TNNT2*, *NKX2-5* (cardiac markers) and *PPARGC1A* (mitochondrial marker)	[123]
hiPSC-CMs (3D microtissues)	Hyperglycemia	High-glucose exposure	➢ Alteration in self-assemble into 3D model and in calcium handling function	[161]
hiPSC-CMs	Hyperglycemia	High-glucose exposure	➢ Pathological hypertrophy ➢ Reduced contractility	[164]
hiPSC-CMs	Insulin resistance	High-palmitate exposure	➢ Oxidation capacity of mitochondria overloaded	[166]
hESC-CMs	Insulin resistance	TNFα and FFA exposure	➢ Increase of *NFKB1*, *IL6* and *CXCL8* (proinflammatory markers) ➢ Inhibition of *PPARGC1A* (mitochondrial marker)	[165]
hESC-CMs	HLHS	Hypoxia for 72 h	➢ Increase of *HIF1α* expression ➢ DNA damage, senescence, reduced cell proliferation and fewer cardiac progenitors	[174]
miPSC-CMs	Hypoxia	Hypoxia for 24 h	➢ Long-term failure contractile phenotype	[173]
P19 ECC derived CMs	Oxidative stress	Different dose-dependent stimuli (e.g., H_2_O_2_)	➢ Impairment of differentiation and contractile phenotype of CMs	[184]
hiPSC-CM	Oxidative stress	Ethanol exposure	➢ Reduction of cell viability, increase of cell loss and overproduction of ROS ➢ Abnormal calcium handling	[195]
mESC-CMs	Toxicity effects	Ethanol exposure	➢ Delay of cardiac differentiation and suppression of Wnt/β-catenin signalling pathway ➢ Suppression of important cardiac transcripts required for the differentiation and maturation	[215]
mESC-CMs	Oxidative stress	Cigarette smoke	➢ Impairment of cardiac-specific genes expression ➢ Pathological heart remodelling	[218]
hESC-CMs	Nicotine toxicity	Nicotine exposure	➢ Reduced viability of hESC ➢ Ca2+ signalling affected in CMs	[212]
hiPSC-CMs	Smoke toxicity	Electronic and regular smoke extract	➢ Slowed beating ➢ Increased ROS and cell death ➢ Genes’ alteration (*MYLK*, *NPPA*, *TNNT2*, *TNNI3*)	[221]
hIPSC-CMs	Hormones	Thyroid and glucocorticoids exposure	➢ Improvement of CMs maturation	[224,225]
mESC-CMs	Chemicals	Flusilazole exposure	➢ Inhibition of cardiac differentiation and changes in CMs gene expression	[234,235]
mESC-CMs	Chemicals	Sparfloxacin and Levofloxacin	➢ Alteration of the frequency and rate of beating of CMs	[236]
hESC-CMs	Chemicals	Trichloroethylene and Perfluorooctane sulfonate	➢ Altered expression of cardiac specific genes ➢ Mitochondrial damage	[237,238]
mESC-CMs	Organic compounds	BPA exposure	➢ Altered CMs morphology ➢ Mitochondrial damage	[240]
hESC-CMs	Organic compounds	BPA exposure	➢ Altered CMs morphology ➢ Higher expression of *NPPA*, *NPPB* ➢ Reduced ATP provision	[241]

## Data Availability

Not applicable.

## Abbreviations

ARE	antioxidant response element
ART	assisted reproductive technologies
ATP	adenosine triphosphate
BMI	Body Mass Index
CHD	congenital heart defects
CMs	cardiomyocytes
CVD	cardiovascular disease
DOHaD	Developmental Origins of Health and Disease
EBs	embryoid bodies
ECC	embryonal carcinoma cells
EZH2	enhancer of zeste homolog 2
eNOS	endothelial nitric oxide synthetase
ESC	embryonic stem cell
FFA	free fatty acids
GDM	gestational diabetes mellitus
HATs	histone acetyltransferases
HDACs	histone deacetylases
hESC-CMs	human embryonic stem cell-derived cardiomyocytes
hESCs	human embryonic stem cells
HFD	high-fat diet
HIFs	hypoxia-inducible factors
HIF1α	hypoxia-inducible factor 1α
hiPSC-CMs	human induced pluripotent stem cell-derived cardiomyocytes
hiPSCs	human induced pluripotent stem cells
HLHS	hypoplastic left heart syndrome
HMOX1	heme oxygenase-1
hPSC	human pluripotent stem cell
hPSC-CMs	human pluripotent stem cell-derived cardiomyocytes
ICSI	intracytoplasmic sperm injection
iPSC	induced pluripotent stem cell
IUGR	intra uterine growth restriction
IVF	in vitro fertilisation
JMJD2A	Jumonji Domain Containing 2A
KEAP1	Kelch-like ECH-associated protein 1
LCFA	long-chain fatty acid
mESC	mouse embryonic stem cell
mESC-CMs	mouse embryonic stem cell-derived cardiomyocytes
miPSC-CMs	mouse induced pluripotent stem cell-derived cardiomyocytes
NCDs	Non-communicable diseases
NFE2L2/NRF2	Nuclear factor erythroid 2-like 2
NO	nitric oxide
NQO1	NAD(P)H quinone oxidoreductase 1
PC	periconceptional
PCR	phosphocreatine
PRC2	polycomb repressive complex 2
PPARs	peroxisome proliferator-activated receptors
PSC-CMs	pluripotent stem cell-derived cardiomyocytes
PSCs	pluripotent stem cells
ROS	reactive oxygen species
SBP	systolic blood pressure
SCs	stem cell
SOD2	superoxide dismutase-2
T1DM	type 1 diabetes mellitus
T2DM	type 2 diabetes mellitus
TGF-β	transforming growth factor-β
WHO	World Health Organization
5hmC	5-hydroxymethylcytosine
5mC	5-methylcytosine
